# Metal-Based Electrocatalysts for Selective Electrochemical Nitrogen Reduction to Ammonia

**DOI:** 10.3390/nano13182580

**Published:** 2023-09-18

**Authors:** Yi-Zhen Zhang, Peng-Hui Li, Yi-Nuo Ren, Yun He, Cheng-Xu Zhang, Jue Hu, Xiao-Qiang Cao, Michael K. H. Leung

**Affiliations:** 1 College of Safety and Environmental Engineering, Shandong University of Science and Technology, Qingdao 266590, China; yizhzhang@sdust.edu.cn (Y.-Z.Z.);; 2 Ability R&D Energy Research Centre, School of Energy and Environment, City University of Hong Kong, Hong Kong, China; 3 School of Chemical and Environmental Engineering, Wuhan Polytechnic University, Wuhan 430024, China; 4 Faculty of Metallurgical and Energy Engineering, Kunming University of Science and Technology, Kunming 650093, China

**Keywords:** electrochemical nitrogen reduction reaction, NH_3_ synthesis, noble-metal-based catalysts, non-noble-metal-based catalysts, metal compound catalysts

## Abstract

Ammonia (NH_3_) plays a significant role in the manufacture of fertilizers, nitrogen-containing chemical production, and hydrogen storage. The electrochemical nitrogen reduction reaction (e-NRR) is an attractive prospect for achieving clean and sustainable NH_3_ production, under mild conditions driven by renewable energy. The sluggish cleavage of N≡N bonds and poor selectivity of e-NRR are the primary challenges for e-NRR, over the competitive hydrogen evolution reaction (HER). The rational design of e-NRR electrocatalysts is of vital significance and should be based on a thorough understanding of the structure–activity relationship and mechanism. Among the various explored e-NRR catalysts, metal-based electrocatalysts have drawn increasing attention due to their remarkable performances. This review highlighted the recent progress and developments in metal-based electrocatalysts for e-NRR. Different kinds of metal-based electrocatalysts used in NH_3_ synthesis (including noble-metal-based catalysts, non-noble-metal-based catalysts, and metal compound catalysts) were introduced. The theoretical screening and the experimental practice of rational metal-based electrocatalyst design with different strategies were systematically summarized. Additionally, the structure–function relationship to improve the NH_3_ yield was evaluated. Finally, current challenges and perspectives of this burgeoning area were provided. The objective of this review is to provide a comprehensive understanding of metal-based e-NRR electrocatalysts with a focus on enhancing their efficiency in the future.

## 1. Introduction

With the ever-increasing global population, pressing worldwide environmental concerns and the challenges in energy supply and conservation, the search for sustainable and eco-friendly energy pathways is strongly demanded to secure our energy future [1,2]. Elemental nitrogen (N_2_) is indispensable to human society and the planet’s ecosystem. Although nitrogen is abundant in the atmosphere (~78% by volume), it cannot be directly utilized by humans. However, nitrogen fixation can convert atmospheric N_2_ to ammonia (NH_3_), which is a more active nitrogen-containing alternative than N_2_. Traditionally, NH_3_ serves as a raw material for the synthesis of fertilizers to sustain the rising global population. NH_3_ is also extensively applied to produce explosives, plastics, resins, pharmaceuticals, and many other chemical compounds for industrial use. Currently, NH_3_ has received considerable attention as a promising carbon-free energy carrier, due to its high hydrogen content (17.65%) and energy density (4.3 kW h kg^−1^), as well as its easy storage in liquids for transportation (9–10 bar) [3,4]. Compared to C-containing fuels, N-containing fuels do not emit CO_2_ upon final decomposition.

Benefiting from one of the most significant scientific inventions in the early 20th century, the Haber–Bosch process (N_2_ + 3H_2_ ⇌ 2NH_3_, Δ_f_H° = −45.940 kJ mol^−1^, Δ_f_G° = −16.407 kJ mol^−1^) is a huge leap towards the mass production of NH_3_ [5,6]. To activate the strong N≡N bonds (46.1 kJ mol^−1^), the Haber–Bosch process requires high temperature (300–500 °C) and high pressure (150–300 atm) with heterogeneous iron-based catalysts. Here, the H_2_ required depends on the carbon-intensive steam reforming of methane, with the input of energy derived from fossil fuels [7]. Consequently, NH_3_ production accounts for 1–2% of the global energy consumption each year and over the 2% of world’s natural gas, giving rise to 3% of energy-related CO_2_ emissions. In this regard, alternative approaches of NH_3_ synthesis need to be developed with relatively low energy consumption, low pollutant production and mild operating conditions. In 2016, the US Department of Energy (DOE) launched the Renewable Energy to Fuels through Utilization of Energy-dense Liquids (REFUEL) program, including NH_3_ as the Carbon-Neutral Liquid Fuels (CNLFs).

In nature, biological N_2_ fixation occurs through multiple proton- and electron-transfer steps relying on the partnership of reductase and nitrogenase enzymes in certain bacteria. Notably, nitrogenase enzymes operate under mild conditions with a significant energy input by the hydrolysis of adenosine triphosphate (ATP) molecules (N_2_ + 6H^+^ + nMg–ATP + 6e^−^ (enzyme) → 2NH_3_ + nMg–ADP + nPi) [8]. Electrochemical catalytic reactions including the hydrogen evolution reaction (HER), oxygen evolution reaction (OER), oxygen reduction reaction (ORR), and carbon dioxide reduction reaction (CO_2_RR) have rapidly developed and achieved excellent results over the past few years [9,10,11,12,13,14]. In addition, nitrate-containing wastewater streams could serve as a nitrogen source via the electrochemical reduction of nitrate into ammonia [15,16,17,18]. Inspired by the biological nitrogen-fixation process, the emerging electrochemical nitrogen-reduction reaction (e-NRR) is promising for achieving NH_3_ production, directly from N_2_ and water under mild conditions with the assistance of renewable energy.

**Figure 1:**
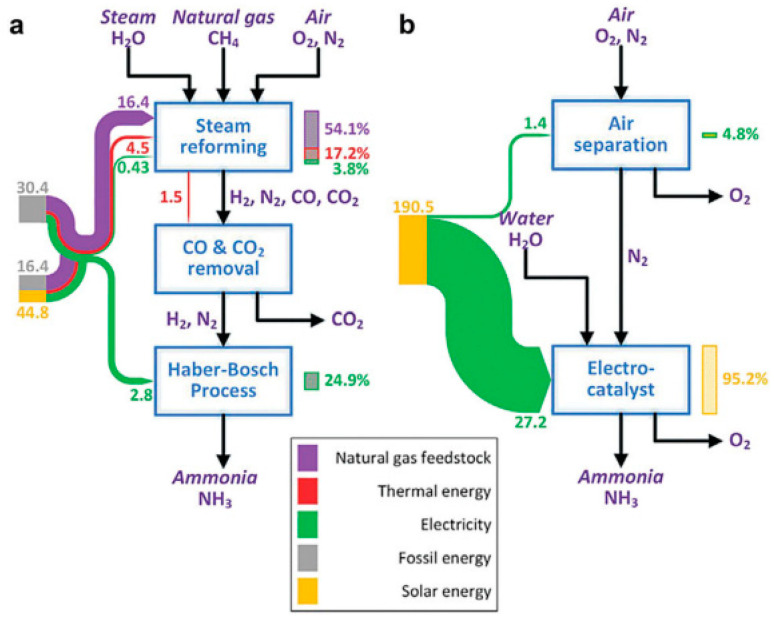
Energy efficiency of NH_3_-synthesis strategies for the (**a**) Haber–Bosch strategy and (**b**) electrocatalytic strategy [19]. Copyright 2018, Elsevier.

Given that steam reforming for hydrogen production accounts for approximately 75% of the energy consumption in the Haber–Bosch process (Figure 1a), the steam-reforming unit with an electrocatalysis unit is a highly effective strategy [19]. Additionally, only two major blocks were observed in this electrocatalytic strategy (Figure 1b). If renewable electricity (from solar energy, wind, etc.) is available, its use in the electrocatalysis strategy will become nearly 100% renewable. In the NH_3_ economy, electrochemical NH_3_ synthesis and NH_3_-powered fuel cells are two critical technologies [20]. The water and nitrogen in air are used as the only reactants to produce NH_3_. The generated NH_3_ can be distributed to users, including farms, NH_3_/H_2_ refuelling stations and residents. The NH_3_-based infrastructure provides a promising way to solve the challenges related to the spatiotemporal fluctuations and the mismatch between supply and demand of electricity. Typically, the detailed cathodic and anodic reactions for e-NRR can be expressed as shown below (Equations (1)–(5)), under different pH aqueous electrolytes [21].

(1) $${\mathrm{Anodic}\;\mathrm{reaction}\;(\mathrm{acidic}\;\mathrm{condition}):\;3H}_{2}O\;\to \;\frac{3}{2}O_{2}{+6H}^{+}{+6e}^{-}\;$$

(2) $${\mathrm{Cathodic}\;\mathrm{reaction}\;(\mathrm{acidic}\;\mathrm{condition}):\;N}_{2}{+6H}^{+}{+6e}^{-}\;\to {\;2\mathrm{NH}}_{3}$$

(3) $${\mathrm{Anodic}\;\mathrm{reaction}\;(\mathrm{basic}\;\mathrm{condition}):\;6\mathrm{OH}}^{-}\;\to \;\frac{3}{2}O_{2}{+3H}_{2}{O+6e}^{-}\;$$

(4) $$\mathrm{Cathodic}\;\mathrm{reaction}\;(\mathrm{basic}\;\mathrm{condition}):N_{2}{+6H}_{2}{O+6e}^{-}\;\to {\;2\mathrm{NH}}_{3}{+6\mathrm{OH}}^{-}$$

(5) $${\mathrm{Overall}\;\mathrm{reaction}:\;2N}_{2}{+6H}_{2}O\;\to {\;4\mathrm{NH}}_{3}{+3O}_{2}$$

Tremendous efforts have been devoted towards the development of e-NRR since 2016, aimed at promoting NH_3_ yield and Faradaic efficiency (FE). However, e-NRR activity is hindered by the poor selectivity of e-NRR and poor activity of current e-NRR electrocatalytic designs. The poor selectivity of e-NRR arises from the competing HER. And the poor catalytic activity is mainly due to the weak affinity of N_2_ to the catalyst surface, which hinders the activation of N_2_ and the corresponding e-NRR efficiency. Accordingly, it is necessary to explore suitable electrocatalysts to overcome these limitations and improve catalytic activity towards e-NRR. Recently, extensive research has focused on designing a suitable electrocatalyst for efficient e-NRR, including noble-metal-based materials, non-noble-metal-based materials, and metal-free materials [2,3,22,23,24].

There are some reviews published elsewhere highlighting the progress of e-NRR electrocatalysts [3,25,26,27,28]. More specifically, Wen et al. [29] summarized the recent progress in low-dimensional nanomaterials with various structures and mentioned the relationship between this structure and e-NRR activities from both theoretical and experimental perspectives. Liu et al. [22] systematically outlined the latest development in novel electrocatalysts, including noble-metal-based catalysts, single-metal-atom catalysts, non-noble metal and their compounds, as well as metal-free catalysts, with various strategies to enhance the e-NRR activities through surface control, defect engineering, and hybridization. From the view of defect engineering, structural manipulation, crystallographic tailoring, and interface regulation, Shi et al. [2] comprehensively summarized the recent development of heterogeneous e-NRR catalysts, together with the catalytic mechanisms, current issues, and critical challenges.

In this review, we begin with the configurations and fundamentals of e-NRR under ambient conditions. Subsequently, the developed e-NRR metal-based catalysts based on noble-metal-based catalysts, non-noble-metal-based catalysts and metal compound catalysts were summarized, from experimental and theoretical perspectives, discussing the structure–function relationship. Finally, current challenges and perspectives of this burgeoning area are provided.

## 2. Configurations of Electrochemical-Reactor for e-NRR

**Figure 2:**
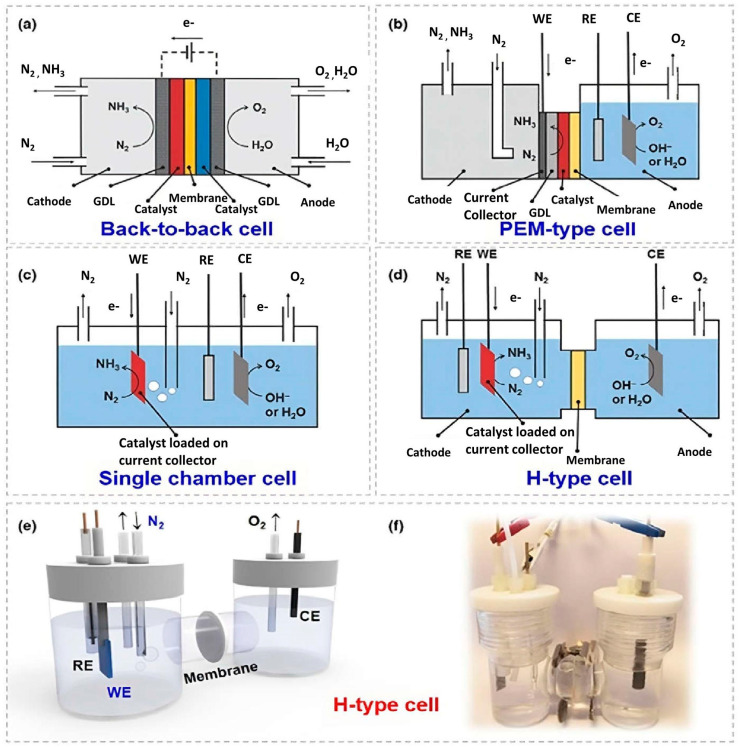
Schematics of different electrochemical-reactor configurations for e-NRR, including (**a**) back-to-back cell, (**b**) proton-exchange membrane (PEM)-type cell, (**c**) single-chamber cell, and (**d**) H-type cell. (**e**,**f**) Schematic and photograph of an H-type cell, including working electrode (WE), reference electrode (RE), counter electrode (CE), and membrane [5]. Copyright 2019, Elsevier.

The electrochemical reactor is important for performing e-NRR. Generally, the configuration of such reactors can be divided into four categories, namely, back-to-back cell, proton-exchange membrane (PEM)-type cell, single-chamber cell, and H-type cell (Figure 2) [30]. 

In the back-to-back cell (Figure 2a), two gas-diffusion electrodes (anode and cathode) are usually separated by a dense membrane, and the anode and cathode are supplied with H_2_O and N_2_ gas, respectively. Among the various types of polymer membranes, perfluorosulfonic acid proton-exchange membranes such as Nafion membranes are widely used [31]. Apart from the use of solid-state electrolytes, liquid electrolytes can also be utilized in back-to-back cells [7].

In the PEM-type cell (Figure 2b), the cathodic chamber is fed with nitrogen gas, and the synthesized NH_3_ gas is directly dissolved in the acidic electrolyte. Different from the back-to-back cell, the anodic chamber in the PEM-type cell is filled with liquid electrolyte, where water is electrolyzed to supply protons for the cathodic reaction [32]. In the back-to-back and PEM-type cells, no direct contact exists between the cathode and electrolyte. Consequently, they possess a favorable advantage for suppressing the HER by limiting the proton supply. However, these two types of cells are not ideal for e-NRR measurements. Several shortcomings including the complex preparation process and the effect from the use of an anion-exchange membrane lead to underestimated NH_3_ production.

In the single-chamber cell (Figure 2c), the anode and cathode are placed in the same chamber without any separator. Nitrogen gas is continuously bubbled into the electrolyte and is subsequently reduced into NH_3_ at the cathode under an applied potential. Simultaneously, OERs occur at the anode. One disadvantage of the single-chamber cell is that the NH_3_ produced from the cathodic reaction may be oxidized at the anode, leading to inaccurate NH_3_ determination. Given that the cathodic and anodic reactions are not separated, gases consisting of NH_3_, hydrogen, and oxygen are produced and discharged from the reactor together at the same time. The dominant HER could also suppress e-NRR at the same region of potential in an aqueous electrolyte-based cell. Consequently, the NH_3_ production rate and FE are limited.

The H-type cell is the most widely studied reactor configuration. The anode and cathode chambers are separated by a membrane to prevent the mixing of products (Figure 2d–f). The working and reference electrodes are located on the same side, attributed to the accurate measurement of the applied potentials and significant decrease in resistance between the working and reference electrodes. In the cathode chamber, nitrogen gas is purged into the electrolyte and is reduced into NH_3_. The anodic reaction is mainly the oxidation of water molecules, also known as the OER. The anodic chamber was sealed without any gas purge in most reported work. Compared with the single-chamber cell, the products in each chamber can be separated in a double-chamber reactor, thereby preventing further oxidation and a mixture of gaseous products. Moreover, different electrolytes can be separated into two chambers so that the cathodic reactions are controlled independently with little influence from the anode. Accordingly, instrumental errors in measurements of e-NRR activity are minimized in the H-type cell compared with other reactors. However, the contribution of NH_3_ dissolved in or leached from the ion-exchange membrane should be carefully handled.

## 3. Fundamental Comprehension on e-NRR

### 3.1. Adsorption of Nitrogen onto the Catalyst Surface

The process of e-NRR includes N_2_ adsorption onto the active sites, activation of N≡N bonds, and a final hydrogenation process. However, the high efficiency of e-NRR is hindered by the difficulty of N_2_ adsorption and activation. Thus, a comprehensive understanding on the dominant reaction pathways is important to design highly efficient electrocatalysts. In the following subsection, an outline of the fundamental comprehension of e-NRR is discussed, with a focus on key steps and dominant reaction pathways.

**Figure 3:**
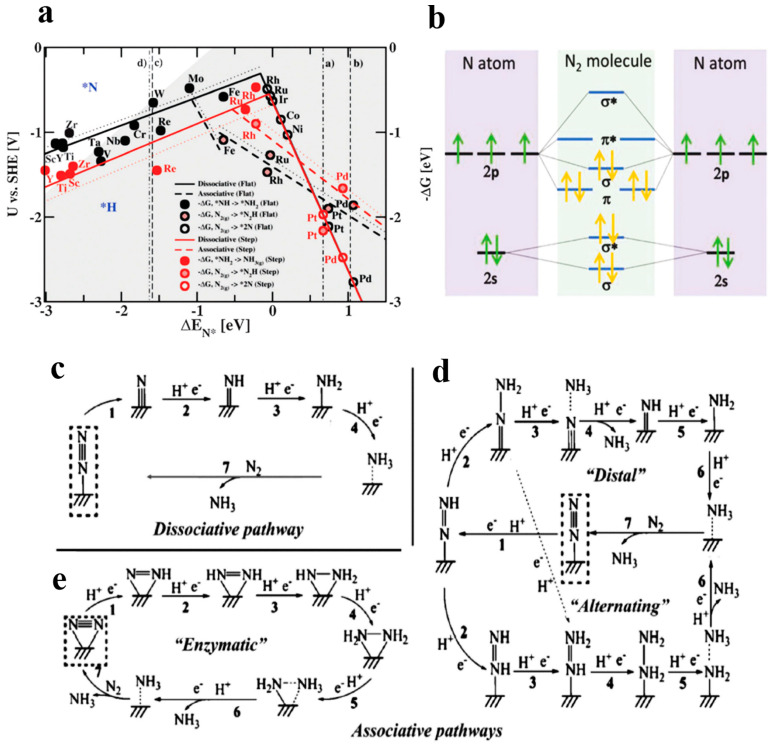
(**a**) Volcano plot of e-NRR for the flat (black lines) and stepped (red lines) transition metal, via dissociative (solid lines) and associative (dotted lines) mechanisms (the redox-potential-limiting step for each metal is highlighted with circles) (an asterisk, *, denotes the adsorption site; the vertical lines (a, b, c, and d) separate different parts and display which species are most strongly bound to the surface) [33], copyright 2012, Royal Society of Chemistry; (**b**) diagrams of N atomic orbitals and their hybridization as N_2_ molecular orbitals [36], copyright 2018, Wiley-VCH; schematic diagram of nitrogen reduction pathway on heterogeneous catalysts [37] ((**c**) dissociative pathway, associative pathways including (**d**) distal and alternating pathway, and (**e**) enzymatic pathway), copyright 2016, American Chemical Society.

The first step is the chemical adsorption of N_2_ onto the catalyst surface. A large amount of N_2_ adsorption sites can be provided by e-NRR catalysts with large surface areas. Accordingly, catalysts with relatively large specific surface areas are promising for the enhancement of e-NRR performance, such as porous-structure materials. The qualitative trends of catalytic activities on different metal surfaces have been summarized by Skúlason and co-workers through theoretical calculations [33]. They assumed that the activation-energy scales with the free energy differed in each elementary step of e-NRR, for the range of flat and stepped transition metals. The key results of this study were illustrated as a volcano plot (Figure 3a), in which the theoretical limiting potentials (U) on different metal surfaces were plotted versus their adsorption energy of N atom (ΔE_N__*_). This plot also showed the relatively limited region in white shading (N-binding), where binding N-adatoms were able to compete with the H-adatoms on the metal surface. With regard to minimizing the parallel HER process, Mo, Fe, Rh, and Ru on top of the volcano diagrams were bound to be the most active surfaces for e-NRR. Metals (Rh, Ru, Ir, Co, Ni, and Pt) on the right legs of the volcano plot were prone to adsorb H-adatoms instead of N-adatoms. In addition, more negative potentials were required for these metals to activate N_2_, resulting in HER that overwhelms e-NRR. Several flat metal surfaces of early transition metals such as Sc, Y, Ti, and Zr, on the left side of the volcano plots, tended to bind N-adatoms more strongly than H-adatoms. However, it remains unclear whether these early transition metals are effective e-NRR electrocatalysts, owing to easy oxidation. As a result, the authors encouraged experimental studies by using some of these metals. Beyond pure metal binding, some nanostructured catalysts with metal–nitrogen bonds have also been found capable of adsorbing N_2_, such as heteroatom-doped carbons (e.g., N/B-doped porous carbon) and metal/nonmetal nitrides (e.g., Mo_2_N, C_3_N_4_) [34]. Therefore, active sites on catalysts can be engineered to preferentially adsorb nitrogen species for e-NRR.

Moreover, the adsorbed amount of N_2_ near the active sites possibly affects e-NRR activity. The solubility of N_2_ gas in water-based electrolytes is about 0.66 mmol/L. Suryanto et al. [35] reported that the use of hydrophobic fluorinated aprotic electrolyte effectively enhanced N_2_ solubility, which could significantly improve the FE of e-NRR. Their results also indicated that the availability of protons was effectively limited and thus suppressed the competing HER. Therefore, the increase in N_2_ solubility near the active sites may be a promising strategy to achieve high e-NRR performance.

### 3.2. Catalytic Activation of N_2_

Due to the high dissociation energy of N≡N bonds (941 kJ mol^−1^) and first-bond cleavage energy (410 kJ mol^−1^), N_2_ is kinetically inert under mild reaction conditions. Activation of N_2_ occurs following the chemical adsorption of N_2_, and is usually considered as one of the rate-limiting steps in e-NRR. The change in the electron density and electron density distribution of the N_2_ molecule can trigger its activation, during adsorption on the catalyst surface. In Figure 3b, an N atom has five valence electrons outside its nucleus, arranged in the configuration 2s^2^2p^3^ [36]. After bonding, the hybridization of the atomic orbitals is divided into four bonding orbitals and four anti-bonding orbitals. Furthermore, there is a large gap (10.82 eV) between the highest occupied molecular orbital (HOMO) with the lowest unoccupied molecular orbital (LUMO), as well as high ionization energy (15.58 eV) that blocks electron transfer [34]. Consequently, N_2_ activation is extremely difficult to achieve under ambient conditions. The first strategy to accelerate N_2_ activation is the improvement of the electron donation and back-donation effect between catalysts and the adsorbed N_2_. Appropriate strong binding on metals, positively charged carbons or vacancies may promote electron transferring from the electrocatalyst matrix to the dinitrogen molecule, therefore accelerating the triple bond activation. Oxygen vacancies in oxides, nitrogen vacancies in nitrides, and surface defects on metals have been further investigated. In another way, it was reported that N_2_ could be activated using the lithium (Li)-mediation assistant method [38]. Li can react directly with N_2_ and dissociate to form Li_3_N. Subsequently, NH_3_ was transformed after the hydrolysis of Li_3_N. However, the procedures of this strategy are relatively complicated.

### 3.3. The Hydrogenation Pathway of N_2_ to NH_3_

Generally speaking, the current reaction mechanisms of the e-NRR on the heterogeneous catalysts are mainly divided into two kinds: the dissociative pathway (Figure 3c), and the associative pathway (Figure 3d,e) [37]. In the dissociative pathway, the triple bonds of an adsorbed nitrogen are firstly cleaved before the hydrogenation reaction, and two independent N atoms subsequently go through a catalytic hydrogenation reaction [39]. The traditional Haber–Bosch process for industrial NH_3_ production mainly follows this mechanism, in which extraordinarily high energy input is required, whereas the process of e-NRR tends to undergo the associative pathway under ambient conditions [40]. In this case, according to the different types of hydrogenation sequences, the associative pathway can be carried out in three possible pathways: the distal, alternating, and enzymatic pathways [30,36]. It is assumed that only one N atom is fixed to the active site, namely, end-on adsorption [41]. In the distal pathway, the N-atom distant from the adsorption site is preferentially hydrogenated continuously. After the release of the first NH_3_, the other N atom bound to the catalyst surface begins to form the second NH_3_, through the hydrogenation process. In contrast, the alternating pathway is to hydrogenate two N atoms in turn, with two NH_3_ molecules generated simultaneously. Instead of an end-on adsorption mode, the enzymatic pathway exhibits side-on adsorption, in which two N- atoms are both adsorbed on the active sites. Additionally, the hydrogenation process involved in the enzymatic path is similar to the alternating pathway. The reduction of nitrogen to NH_3_ undergoes these possible mechanisms, resulting in different intermediates, such as diazene (N_2_H_2_), NH_3_, and hydrazine (N_2_H_4_).

However, apart from the difficulty of nitrogen activation, the e-NRR in aqueous solution is limited by the competition of HER [42]. Several processes at the electrode–electrolyte interface occur concurrently, involving the diffusion and adsorption of reactant species, transfer of electrons and protons, as well as desorption of species, where e-NRR and HER share some reaction species for basically electro-hydrogenation reactions [43,44]. Moreover, the standard equilibrium potential of HER (E^0^ = 0 V, vs. RHE) is similar to that of e-NRR (E^0^ = 0.092 V, vs. RHE), but HER has much faster reaction kinetics [36]. As a result, e-NRR typically suffers from a low reaction rate and low selectivity (FE) for NH_3_ production. The competition between HER and e-NRR can be controlled by optimizing the electrolyte and potential, the local availability of protons and N_2_ molecules near the catalyst, and revealing the relationships between structure and activity for rational catalyst design.

## 4. Advances in Metal Catalysts Design for e-NRR

In recent years, significant efforts have been devoted to design and fabricate an efficient electrocatalyst for NH_3_ production [45]. In view of the different compositions and characteristics, the various e-NRR metal catalysts can be classified into metal-based materials and metal compound materials. The recent progress of reported e-NRR metal catalysts is summarized and discussed, with a particular emphasis on their e-NRR performance and catalytic reaction.

### 4.1. Metal-Based Catalysts

#### 4.1.1. Noble-Metal-Based Catalysts (Ru, Rh, Pt, Au, and Pd)

Noble metal catalysts have been proved as promising electrocatalysts in plenty of electrochemical reactions (such as HER, OER, and ORR), due to their marvelous conductivity, active polycrystalline surfaces, and appropriate adsorption of various reactants. Recently, noble metal catalysts such as Pt, Au, Ag, Ru, and Rh, have been explored for e-NRR to NH_3_ synthesis.

**Figure 4:**
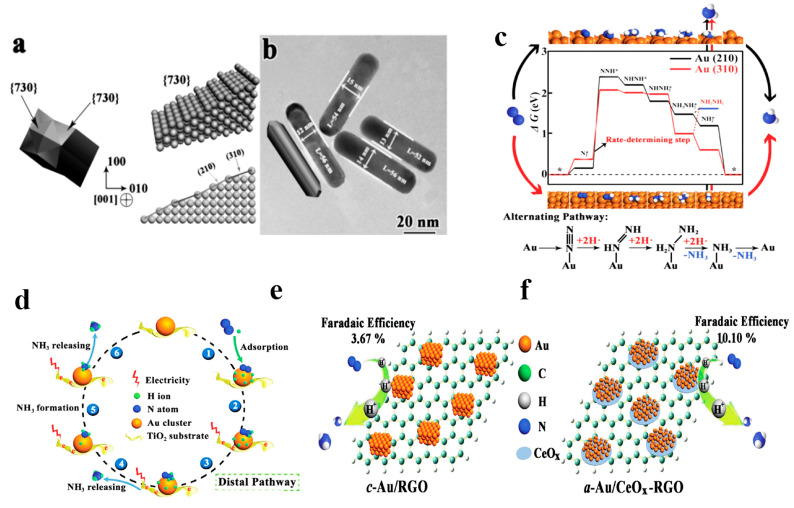
(**a**) Geometric models of Au THH NR and exposed facet [47]; (**b**) TEM images of Au THH NRs [47]; (**c**) free energy diagram and alternating hydriding pathway for e-NRR on Au (210) and Au (310) at equilibrium potential (* represents the adsorption site) [47], copyright 2016, WILEY-VCH Verlag GmbH & Co. KGaA, Weinheim; (**d**) proposed pathway for the NH_3_ synthesis using Au/TiO_2_ catalyst [51], copyright 2017, WILEY-VCH Verlag GmbH & Co. KGaA, Weinheim; schematic illustration of e-NRR by catalysts of (**e**) c-Au/RGO and (**f**) a-Au/CeOx–RGO [52], copyright 2017, WILEY-VCH Verlag GmbH & Co. KGaA, Weinheim.

Au electrocatalysts have been studied as the most promising noble catalysts for e-NRR [46], by controlling the morphology-dependent effect and metal–support synergetic effect. The former involves the creation of additional active sites by controlling morphology, crystal facets orientation, and crystallinity. As shown in Figure 4, Yan et al. [47] synthesized tetra-hexahedral Au nanorods (Au THH NRs) as heterogeneous electrocatalysts and characterized e-NRR activity in an N_2_-saturated 0.10 M KOH solution. The measured angle implied that the bevels on the THH Au NR were high-index (730) planes, composed of the (210) and (310) sub-facets (Figure 4a,b). A large number of active sites can be provided to capture and activate N_2_, due to the exposed high-index (210) and (310) facets. The Au THH NRs endowed a highest NH_3_ production rate of 1.648 µg h^−1^ cm^−2^ and maximum FE of 4.02% at −0.20 V vs. reversible hydrogen electrode (RHE). The density functional theory (DFT) calculations predicted that the e-NRR process preferably follows the alternating pathway, with the rate-determining step of N_2_ dissociation for both Au (210) and Au (310) (Figure 4c). Wang et al. [48] reported a rapid approach for the fabrication of flower-like Au microstructures (Au flowers). In a comparative experiment, the e-NRR performance of Au flowers (NH_3_ rate: 25.57 μg h^−1^ mg_cat._^−1^, FE: 6.05%) outperformed the Au sphere counterpart. It indicated that Au flowers (particle size: ~900 nm) with highly dendritic structures could provide abundant electrocatalytically active sites, and therefore, promote e-NRR activity. The use of hollow gold nanocages (Au HNCs) as an effective electrocatalyst was also evaluated for e-NRR in 0.50 M LiClO_4_ [49]. The highest FE of Au HNCs (30.2%) was achieved at −0.40 V vs. RHE, while the maximum NH_3_ production (3.90 µg h^−1^ cm^−2^) was obtained at −0.50 V vs. RHE. In contrast experiments, the e-NRR activity of Au HNCs was much better than other Au nanoparticles of various shapes (i.e., Au nanorods, Au nanospheres, and Au nanocubes) in the same conditions, resulting from the increased surface area and confinement effects.

Unlike the morphology-dependent effect, the metal–support synergetic effect contributes to improving the intrinsic activity of active centers. Therefore, the combination of the metal with support as composite may present a new route for reducing the usage of noble metal. For example, Zhao et al. [50] reported a nano-gold catalyst supported on a boron organic polymer (Au/M-BOP) as electrocatalyst for electrochemical reduction from N_2_ to NH_3_. Yan and co-workers [51] studied the Au/TiO_2_ catalyst as a heterogeneous catalyst for e-NRR, synthesized using Au sub-nanoclusters (~0.50 nm) embedded in commercial TiO_2_ support. Unexpectedly, the obtained Au/TiO_2_ endowed the e-NRR with a high yield (NH_3_: 21.4 µg h^−1^ mg^−1^, FE: 8.11%) at −0.20 V vs. RHE. Moreover, it should be noted that the apparent catalytic activity decreased after tuning particle size of Au species dispersed on TiO_2_ ranging from nanometer down to sub-nanometer sizes. This work also indicated that the isolated precious metal onto oxide supports provided a well-defined system. The proposed pathway for the NH_3_ synthesis using Au/TiO_2_ catalyst was shown in Figure 4d, displaying a distal hydriding pathway.

This group continued to explore the effectiveness of Au and proposed CeO_x_-induced amorphous Au nanoparticles on reduced graphite oxide (a-Au/CeO_x_-rGO) as e-NRR electrocatalysts [52]. As shown in Figure 4e,f, it was found that the CeO_x_ played an important role in transferring the crystalline Au NPs into the amorphous ones. Compared with its crystalline counterpart, a-Au/CeO_x_-rGO achieved a higher 10.10% FE with an NH_3_ yield of 8.3 µg h^−1^ mg_cat._^−1^ at −0.20 V vs. RHE, because of their higher concentration of active sites and more structural distortion. In another study, Wang and co-workers [53] reported the Au/N-doped nano-porous graphitic carbon membrane (NCM) electrocatalyst. The synergistic effect between NCM and Au promoted the N_2_ adsorption and thereby improved the conversion of N_2_ to NH_3_.

**Figure 5:**
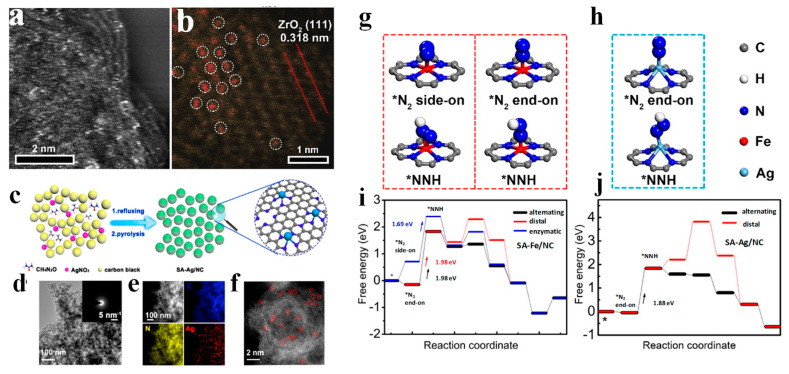
(**a**,**b**) HAADF-STEM image of the Ru@ZrO_2_/NC [55], copyright 2018, Elsevier Inc. (**c**–**f**) Synthesis and structural characterization of SA-Ag/NC [57]: (**c**) schematic illustration; (**d**) TEM image; (**e**) HAADF-STEM images and corresponding element maps; (**f**) aberration-corrected HAADF-STEM image); (**g**–**j**) DFT computation results of e-NRR on SA-Fe/NC and SA-Ag/NC [57], copyright 2020, American Chemical Society.

The precious metal Ru is also a hot research subject in the electroreduction of N_2_. Similarly, the crystal structure and the particle size of Ru also have a great influence on e-NRR activity. Wang et al. [54] studied Ru nanoparticles as e-NRR electrocatalysts in 0.01 M HCl aqueous solution. The maximum yield rate of 5.50 mg h^−1^ m^−2^ was achieved at −0.10 V vs. RHE, whereas the highest FE was 5.40% at 0.10 V vs. RHE. The DFT calculations indicated that the efficient e-NRR activity at the low overpotential was attributed to instantaneous N_2_ adsorption on Ru (001) surfaces and the spontaneous hydrogenation process by a dissociative mechanism. In another study, isolating Ru single atoms in N-doped porous carbon as electrocatalyst greatly promoted N_2_-to-NH_3_ conversion (Figure 5a,b), affording an NH_3_ formation rate of 3.665 mg h^−1^ mg _Ru_^−1^ at −0.21 V vs. RHE [55]. It was found that the addition of ZrO_2_ can effectively suppress the competitive HER, reaching a high FE of 21% at a low overpotential. From calculation results, the e-NRR mainly occurred at Ru sites with O vacancies, which was permitted through the stabilization of *NNH (low overpotential), destabilization of *H (high e-NRR/HER selectivity), and enchantment of N_2_ adsorption (to initiate the e-NRR process).

In addition to the above catalysts, Rh, Ag, and Pd have also been studied for e-NRR, due to their strong adsorption energy and low overpotentials [33]. Surfactant-free atomically ultrathin Rh nanosheets (Rh NSs) were synthesized and used as an effective e-NRR in a 0.10 M KOH solution [54]. Benefiting from their unique ultrathin two-dimensional (2D) structure with abundant surface and modified electronic structure, Rh NSs exhibited an excellent e-NRR performance with a high NH_3_ yield rate (23.88 µg h^−1^ mg_cat._^−1^) and selectivity (no N_2_H_4_ generation) at −0.20 V vs. RHE. But, the FE at the same potential was only 0.217%, due to the dominant HER process. Yin et al. [56] reported Ag triangular nanoplates (Ag TPs) as e-NRR catalysts with efficient activity of NH_3_ generation. The e-NRR activity of Ag TPs was much more efficient than circular Ag nanoparticles, owing to the more anchored atoms at sharp edges and corners on Ag TPs. Single Ag sites with the Ag-N_4_ coordination (SA-Ag/NC) were synthesized massively by targeting the admolecules (Figure 5c), confirming that abundant Ag SAs exist in the carbon matrix by TEM and HAADF-STEM (Figure 5d–f). SA-Ag/NC achieved a record-high NH_3_ yield rate (270.9 μg h^−1^ mg_cat._^−1^ or 69.4 mg h^−1^ mg Ag^−1^) and a desirable FE (21.9%) in HCl aqueous solution [57]. Through 20 consecutive cycle tests, the stability of SA-Ag/NC was maintained. Furthermore, to eliminate or quantify the sources of contamination, a rigorous reduction experiment was recommended by the isotopic labeling experiment using ^15^N_2_, reliably confirming the ammonia production only from the N_2_ source [4,58]. As expected, through the isotopic labeling experiment, the NH_3_ generation was verified from the gaseous N_2_ over SA-Ag/NC during the e-NRR process. Based on first principles calculations (Figure 5g–j), the emergence of vertical end-on *N_2_ and oblique end-on *NNH admolecules on single metal sites in succession were energetically favorable for e-NRR.

From the point of view of morphology control, crystallographic tailoring, structural manipulation, and defect engineering, the noble metal catalysts were summarized, aiming to enhance the e-NRR activity. Combined with discussing the relationship between the structure and e-NRR activity based on experimental and theoretical results, they are expected to provide a reference for the rational design of e-NRR electrocatalysts in a targeted manner.

#### 4.1.2. Non-Noble-Metal-Based Catalysts

There is a growing desire to explore resource-rich metal in the earth, in order to reduce cost and improve the applicability of e-NRR technology. Due to the important role of the FeMo cofactor in biological nitrogen fixation and Fe-based catalysts in Haber–Bosch technology, Mo- and Fe-based electrocatalysts have been explored toward e-NRR.

**Figure 6:**
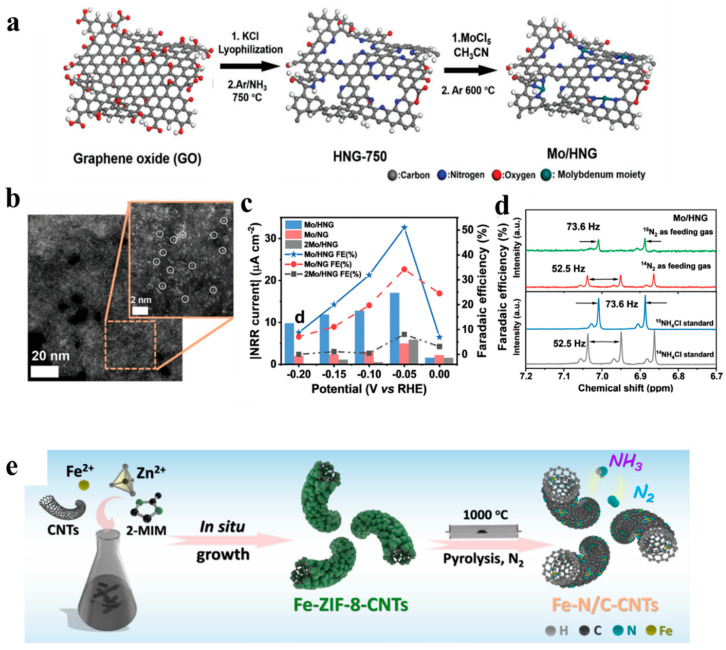
(**a**) Schematic illustration of the synthetic process for the Mo/HNG catalyst [62], (**b**) atomic resolution HAADF-STEM images of Mo-HNG and magnified area with circled individual Mo atoms anchored on the carbon matrix at N-rich edges [62], (**c**) NH_3_ yields, FEs and partial current densities for e-NRR on Mo/HNG, Mo/NG, 2Mo/HNG catalysts determined from chronoamperometric measurements [62], (**d**) 1H NMR spectra of the resultant electrolyte obtained from the e-NRR measurement of Mo/HNG, respectively, using ^14^N_2_ or ^15^N_2_ as the isotopic nitrogen source at −0.05 V [62], copyright 2022, Wiley-VCH GmbH. (**e**) Schematic illustration of the synthesis of Fe−N/C−CNTs [67], copyright 2018, American Chemical Society.

To explore the effect of crystal phase orientations for Mo catalysts, Yang et al. [59] prepared four kinds of Mo-based nanofilms with different facet orientations and surface morphology. Mo (110) plane can adsorb N adatoms more strongly than H adatoms, while Mo (211) dominantly follows competitive HER. As a result, Mo (110) was more efficient with FE of 0.72% at a low overpotential of −0.49 V vs. RHE. This study showed that morphology control was also a feasible way to improve the catalytic activity of pure non-noble metal toward e-NRR. A study of DFT simulation predicted that single Mo atom fixed on a defective boron nitride (BN) monolayer could be potentially used as a N_2_ fixation electrocatalyst, where dispersed Mo atoms bonded to N atoms contributed to activate N_2_ molecules, selectively stabilize N_2_H*, or destabilize NH_2_* during e-NRR [60]. Based on this study, Han et al. [61] reported single Mo atoms anchored onto N-doped porous carbon (SA-Mo/NPC) as e-NRR electrocatalysts. Benefiting from the optimized abundance of active sites and 3D hierarchically porous carbon frameworks, SA-Mo/NPC achieved a high NH_3_ yield rate (34.0 ± 3.6 μg h^−1^ mg_cat._^−1^) and a high FE (14.6 ± 1.6%) in 0.10 M KOH electrolyte at −0.30 V vs. RHE. Similarly, efficient e-NRR activity and durability were also obtained by SA-Mo/NPC in 0.10 M HCl acid electrolyte. The authors also concluded that Mo–N sites of atomically dispersed Mo atoms bonding to N were the catalytic active sites. As shown in Figure 6a,b, the stabilized single Mo atoms anchored on holey N-doped graphene (Mo/HNG), with a continuous porous skeleton and plenty of edges containing N-coordination sites, were synthesized through a potassium salt-assisted activation process [62]. As plotted in Figure 6c, at −0.05 V vs. RHE, Mo/HNG exhibited an exceptional FE of 50.2% for NH_3_ production (partial reduction current density: 17.0 µA cm^−2^) and a NH_3_ production yield rate of 3.6 µg h^−1^ mg_cat_^−1^. During continuous electrolysis (20,000 s), Mo/HNG still maintained over 50% FE (−0.05 V vs. RHE), with only 0.0125% of Mo on the electrode dissolved (ICP-MS test), exhibiting good stability. The isotopic labeling experiments were measured, respectively, using abundant natural ^14^N_2_ and ^15^N_2_ as feed gas [63]. As shown in Figure 6d, the NH_4_^+^ splitting patterns in 1H nuclear magnetic resonance were consistent with the corresponding resultant electrolyte using isotopic ^14^N_2_ or ^15^N_2_ source, presenting a specific double peak for ^15^NH_4_^+^ and three peaks for ^14^NH_4_^+^ [64]. Through theoretical calculations, it is unveiled that the edge coordinated Mo atoms and the existence of vacancies on holey graphene jointly contribute to the intriguing e-NRR activity.

As one of the most earth-abundant metals, Fe-based catalysts have also shown great potential as excellent e-NRR electrocatalysts. For instance, Wang et al. [65] theoretically proposed the catalytic mechanisms of single Fe atom embedded N-doped graphene for e-NRR. The results indicated that the magnetic moment of the Fe atom increased with the increase in coordination of the neighboring N atom, resulting in a lower overpotential of N_2_ reduction. In experiment, Wang et al. [66] recently used a single-atom catalyst (iron on N-doped carbon, Fe_SA_-N-C) as an e-NRR electrocatalyst, enabling a dramatically enhanced FE. Here, the DFT calculations suggested that the Fe_SA_-N-C structure could effectively attract the access of N_2_ molecules with a small energy barrier, which benefits preferential N_2_ adsorption instead of H adsorption. The isotope-labeling experiments and control experiments indicated that the generated NH_3_ entirely comes from the e-NRR process catalyzed by Fe_SA_-N-C. Careful characterization and consecutive recycling electrolysis were preformed, suggesting its excellent stability. In another study, an Fe-N/C-carbon nanotube catalyst (Fe-N/C-CNTs) was designed, through carbonizing a metal–organic framework and carbon-nanotube-based composite [67], with built-in Fe−N_3_ sites. The corresponding synthesis process was shown in Figure 6e. In 0.10 M KOH electrolyte, the optimal NH_3_ formation rate was 34.83 μg h^−1^ mg_cat._^−1^ with an FE of 9.28% at −0.20 V vs. RHE. The favorable e-NRR activity was attributed to Fe−N_3_ species as active sites. The theoretical results further revealed that the e-NRR reaction proceeded preferentially via the distal pathway.

Recently, Leung’s group was dedicated to non-noble bimetals on nitrogen-doped carbons, selecting from either side of the theoretical volcano plot for the e-NRR. Dispersed Mo-Co bimetallic nanoparticles immobilized on N-doped porous carbon (Mo-Co/NC) were developed and exhibited the enhanced activity and selectivity of e-NRR electrocatalysis with an ammonia yield, in comparison to single-metallic Co/NC [68]. Additionally, to overcome the sluggish kinetics of the proton-coupled electron transfer on the single-atom site, Leung et al. [69] synthesized atomically dispersed Co-Mo pairs anchored on N-doped carbon frameworks (Co-Mo-SA/NC) through calcinating Co–Mo-doped zinc-based zeolite imidazole framework precursors. Revealed by Bader charge analysis and charge density difference analysis, 0.35 e^−^ and 0.30 e^−^ were, respectively, transferred to N1 on the Mo-end and N_2_ on the Co-end from Co–Mo active sites; simultaneously, Co and Mo atoms with two occupied d orbitals possess the capability to donate their electrons to the empty p* orbital of N, ultimately forming triple bonds. Nevertheless, the N_2_ on a single active site follows the electron acceptance–donation concept, resulting in a significant increase in energy required for the initial activation of N_2_. Consequently, the Co–Mo-SA/NC catalyst achieves outstanding e-NRR performance in 0.1 M Na_2_SO_4_ solution with 37.73 μg h^−1^ mg_cat._^−1^ and a desirable FE of 23.18% at −0.1 V vs. RHE, which are twofold higher than those of the isolated single-atom Co (Co-SA/NC) or Mo (Mo-SA/NC) catalyst.

Apart from the aforementioned Fe- and Mo-based catalyst, other transition metals have also emerged as electrocatalysts for N_2_ fixation [70,71,72,73]. For example, Co single-atom-embedded N-doped porous carbon (CSA/NPC) was synthesized as an electrocatalyst for e-NRR [74]. At a low overpotential of −0.20 V, CSA/NPC presented a high NH_3_ yield rate of 0.86 mmol cm^−2^ h^−1^ and a FE of 10.50%, attributed to the positive effects of Co single atoms, N-doping, and porous structure. In another work, Wang et al. [75] reported atomically dispersed Ni sites on a carbon framework with nitrogen-vacancy (Ni_x_-N-C) as an effective non-noble-metal electrocatalyst for the e-NRR, synthesized from a Ni-doped ZIF-8 precursor. Compared with Ni clusters supported on the N-doped carbon framework, significant e-NRR activity was observed on Ni_x_-N-C with an NH_3_ production rate of 115 µg cm^−2^ h^−1^ at −0.80 V (vs. RHE) and FE of 20% at −0.60 V (vs. RHE) in LiClO_4_ solution. From calculation results, Ni-N_x_ sites were responsible for the experimentally observed activity and the potential determining step was the hydrogenation during the e-NRR. Cu as a common and low-cost metal has also been studied for e-NRR by Zang and co-workers [76]. In this experiment, a Cu single atom on a porous N-doped carbon network (NC-Cu SA) was studied for catalytic performance toward e-NRR in both alkaline and acidic solutions. The NC-Cu SA exhibited a high NH_3_ yield rate and FE, specifically ~53.30 μg h^−1^ mg_cat._^−1^ and 13.80% under 0.10 M KOH, ~49.30 μg h^−1^ mg_cat._^−1^ and 11.70% under 0.10 M HCl. Similarly, the experimental analysis and DFT calculations indicated that the local Cu−N_2_ coordination was identified as the efficient sites and responsible for the outstanding e-NRR performance.

Up to now, non-noble-metal-based materials have been reported as efficient e-NRR electrocatalysts, most investigations mainly focused on their composites including nitrides, carbides and oxides. More discussion about metal compounds will be summarized in the following section.

### 4.2. Metal Compound Catalysts

#### 4.2.1. Metal Sulfide and Metal Nitride Catalysts

In recent years, a range of metal sulfides and metal nitrides have been taken into consideration for e-NRR. Although the intrinsic catalytic activity of MoS_2_ for water reduction suppressed the e-NRR process, MoS_2_ is still considered and utilized as an electrocatalyst to catalyze the N_2_ reduction reaction. Sun’s group theoretically predicted and experimentally confirmed that MoS_2_ as an active e-NRR electrocatalyst achieved a high NH_3_ yield rate (8.08 × 10^−11^ mol s^−1^ cm^−1^) and FE (1.17%) at −0.50 V vs. RHE [77]. Impressively, this study further indicated that MoS_2_ was still active for e-NRR, where a strong HER occurs. Soon thereafter, they found that defect-rich MoS_2_ (DR MoS_2_) nanoflower could greatly boost electrocatalytic N_2_ reduction to NH_3_^39^. Compared with the defect-free counterpart, a high FE (8.34%) and high NH_3_ yield (29.28 µg h^−1^ mg_cat._^−1^) were obtained at −0.40 V vs. RHE. DFT calculations revealed that the potential-determining step was *NH_2_ → *NH_3_, and the barrier of DR MoS_2_ (0.60 eV) was lower than the barrier of the defect-free catalyst (0.68 eV). In another theoretical study, Fe-doped MoS_2_ through an associative distal pathway revealed that the presence of a vicinal Fe atom enabled highly selective chemisorption of N_2_, which was conducive to the efficient activation of the N≡N bonds [78]. This investigation provides some new ideas for designing active metal sulfides for the electrochemical synthesis of NH_3_.

Under the comprehensive theoretical investigation on a range of transition metal nitrides (TMN) for e-NRR, metal nitrides are believed to offer the potential advantages for N_2_ fixation [79]. Skúlason et al. [80] studied the possibility of nitrogen activation for electrochemical NH_3_ formation on a range of (111) TMN surfaces (ScN, TiN, VN, CrN, MnN, YN, ZrN, NbN, MoN, HfN, TaN, WN, and ReN). It was found that VN, CrN and MnN were the most promising candidates, which were expected to catalyze e-NRR at the relatively low onset potential (from −0.80 V to −0.50 V vs. SHE). However, the possibility of poisoning toward MnN and WN was found in an electrochemical environment. Only NbN with the (111) plane can be regenerated itself and can activate N_2_ to NH_3_, with active and stable activity.

To date, only Mo- and V-based nitrides have been experimentally studied and proved to enable efficient catalytic activity toward e-NRR. Based on the theoretical investigations, vanadium nitride nanosheet [81], vanadium nitride nanowire array [82], and vanadium nitride nanoparticles [83] have been fabricated and tested for e-NRR activity. Comparatively, vanadium nitride (VN) nanoparticles exhibited better catalytic performance for e-NRR, with an NH_3_ production rate and FE of 3.30 × 10^−10^ mol s^−1^ cm^−2^ and 6.0%, respectively [83]. According to a combination of ex situ and operando characterizations, multiple vanadium oxide, oxynitride and nitride species were present on the surface. Among them, VN_0.7_O_0.45_ was identified as the active phase in the e-NRR, and the conversion of VN_0.7_O_0.45_ to VN phase was proposed as the deactivation pathway.

Except for vanadium nitride, concerns regarding molybdenum nitride have been recently discussed. Li et al. [84] theoretically studied the 2D layered molybdenum nitride nanosheets (MoN_2_) as NH_3_ synthesis catalysts at room temperature. According to calculations, MoN_2_ exhibited excellent performance for adsorption and activation of N_2_ molecules, but large energy input was requested to regenerate the MoN_2_ surface. However, the e-NRR performance can be remarkably promoted after Fe-doping, with ΔG_max_ = 0.47 eV for the rate-determining step. The conclusion about Fe-doping agreed with the recent report regarding Fe-doped MoS_2_ [78]. Experimentally, the MoN nanosheet array on a carbon cloth (MoN NA/CC) was explored as a high-performance catalyst towards e-NRR in 0.10 M HCl under ambient conditions [81]. This catalyst achieved an NH_3_ yield of 3.01 × 10^−10^ mo1 s^−1^ cm^−2^ and an FE of 1.15% at −0.30 V vs. RHE. Moreover, N_2_H_4_ was not detected, and therefore, MoN NA/CC showed excellent selectivity to NH_3_. The potential-determining step of this catalyst was the second protonation of the surface N, confirmed by DFT calculations. In another study, this group reported a Mo_2_N nanorod as an efficient electrocatalyst to electrochemically convert N_2_ to NH_3_ [85]. Mo_2_N nanorods were prepared by nitriding of the precursor MoO_2_ in an NH_3_ atmosphere. Compared with MoO_2_, the NH_3_ yield of Mo_2_N was much higher. When tested in 0.10 M HCl, Mo_2_N could enhance the FE to 4.5% at an applied potential of −0.30 V vs. RHE, which was higher than MoN NA/CC in the previous report. DFT calculations also confirmed that the free energy barrier of the potential-determining step for the Mo_2_N catalyst was dramatically lower than MoO_2_. Based on the studies above, the FE in e-NRR still needs to be further improved in the future.

#### 4.2.2. Metal Carbide Catalysts

The metal carbides are an interesting class of catalysts. According to the d orbital theory, transition metal carbides with unoccupied d orbitals should have good adsorption ability for electron-enriched reactants [86,87]. In order to investigate the viability of using molybdenum carbide as an e-NRR electrocatalyst, a computational study was conducted by Matanovic and co-workers [88]. The comparison between two competing reactions (HER and NRR) revealed that MoC (111) was the only surface that suppressed the adsorption of H-atoms at low overpotentials, among various crystallographic surfaces. Additionally, the e-NRR in MoC (111) surface could take place at small negative potentials of −0.30 V vs. SHE, and followed an associative reaction pathway. The authors also illustrated that introducing carbon vacancies could mitigate hydrogen evolution and H-adatom accumulation. Recently, molybdenum carbide nanodots embedded in ultrathin carbon nanosheets (Mo_2_C/C) were designed by molten salt synthesis, and used as a catalyst candidate for e-NRR [89]. The obtained Mo_2_C/C nanosheets exhibited efficient e-NRR catalytic activity with an NH_3_ production rate of 11.3 µg h^−1^ mg^−1^ and FE of 7.8%. Based on the experiments and DFT calculations, the catalytic active center of Mo_2_C nanodots was favorable for adsorbing N_2_, and their unique electronic structure was feasible for N_2_ activation and hydrogenation. MoS_2_, MoO_3_, MoN and Mo_2_N have been reported as e-NRR electrocatalysts with relatively lower FE of 1.17%, 1.9%, 1.15%, and 4.5% [77,81,85,90], respectively. To continuously enhance the performance, Sun’s group reported Mo_2_C nanorod as a catalyst for electrocatalytic N_2_ reduction to NH_3_ production [90]. At the applied potential of −0.30 V vs. RHE, such a catalyst achieved a high FE of 8.13% and NH_3_ yield rate of 95.10 μg h^−1^ mg_cat._^−1^ in 0.10 M HCl electrolyte. To date, metal carbide nanocomposites such as the e-NRR catalyst are rarely reported.

**Figure 7:**
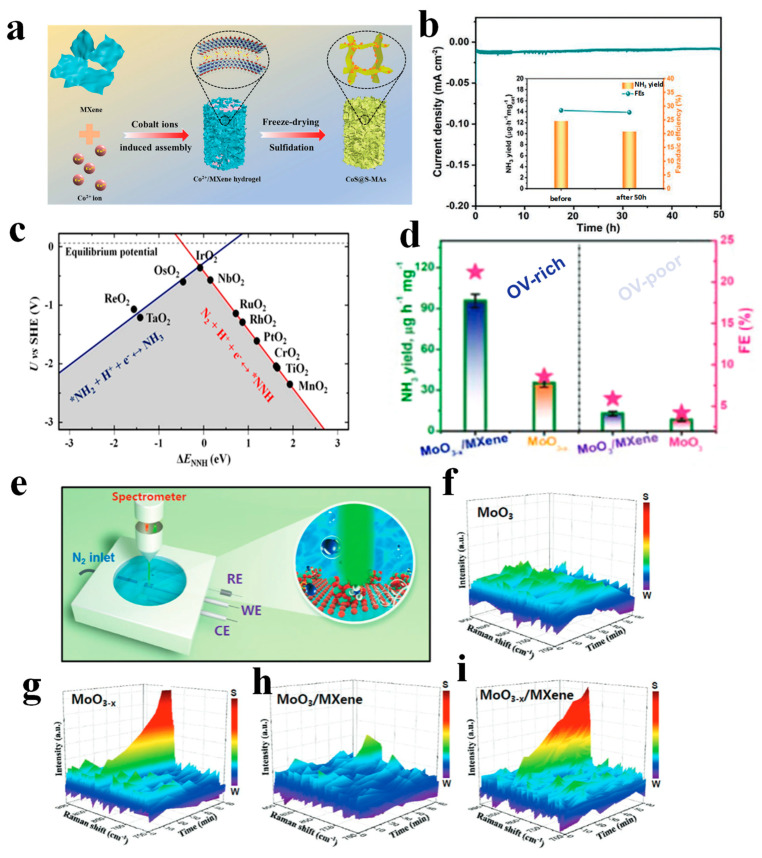
(**a**) Schematic illustration of the synthesis of CoS@S-MAs. (**b**) Chronoamperometry curve of CoS@S-MAs for 50 h electrolysis (−0.15 V vs. RHE), and corresponding NH_3_ yield and FEs before and after 50 h (inset), Copyright 2021, Wiley-VCH GmbH. (**c**) Volcano plot of plotting the predicted onset potentials for e-NRR on the (110) facet of transition-metal dioxides against the binding energy of NNH, ΔE_NNH_, as the descriptor of catalytic activity [96]. Copyright 2017, American Chemical Society; (**d**) NH_3_ yields/FEs at −0.4 V of MoO_3_, MoO_3-x_, MoO_3_/MXene and MoO_3-x_/MXene (pink star represents FE, dotted line separates OV-rich materials and OV-poor materials), (**e**) schematic of tailor-made electrolytic cell, (**f**–**i**) 3D plots of the time-dependent in situ Raman spectroscopy of different catalysts for e-NRR process at −0.4 V [97], Copyright 2021, Wiley-VCH GmbH.

MXenes, a group of 2D layers of transition metal carbides, are promising catalysts for e-NRR [91,92]. A large number of studies have focused on theoretical calculations. In 2019, Luo et al. [93] firstly reported that the MXene (Ti_3_C_2_T_x_) nanosheets attached to a vertically aligned metal host could achieve a high NH_3_ FE (5.78%) at an ultralow overpotential of −0.10 V vs. RHE. From the combined experimental and theoretic results, a greater number of exposed edge sites and a metal host with poor HER activity were responsible for higher e-NRR activity. In another work, a Ti_3_C_2_T_x_ MXene nanosheet was used as both a conductive and Ti source toward the in situ hydrothermal growth of TiO_2_ nanoparticles [94]. The combination of TiO_2_ and Ti_3_C_2_T_x_ led to a synergistically active Ti-based nanohybrid catalyst with enhanced activity. As a result, such a TiO_2_/Ti_3_C_2_T_x_ hybrid catalyst exhibited an NH_3_ yield of 26.32 μg h^−1^ mg_cat._^−1^ with an FE of 8.42% in 0.10 M HCl electrolyte (−0.60 V vs. RHE). It is universally known that 3D porous MXene-based aerogel architectures are beneficial for rapid mass diffusion, higher exposure of electrochemically active sites, and faster mass diffusion and charge/electron transport. Herein, Li et al. [95] designed a functional 3D MXene-based composite heterojunction aerogel (MS@S-MAs) for e-NRR, fabricating metal sulfide nanoparticles confined in 3D S-doped MXene sheets (Figure 7a), via divalent metal-ion-induced assembly following the thermal sulfidation method. Remarkably, CoS@S-MAs gave the best reactivity among metal sulfide nanoparticles (M = Co, Fe, Cu, Ni), showing an NH_3_ yield rate and a FE of 12.4 μg h^−1^ mg_cat._^−1^ and 27.05% at the lower potential of −0.15 V vs. RHE in Na_2_SO_4_ solution. Additionally, CoS@S-MAs, after 50 h of e-NRR, displayed a slight loss in FE and NH_3_ yield rate (Figure 7b), indicating the excellent long-term stability of the catalyst. This study offers a new prospect for 3D porous aerogel materials for application in e-NRR metal oxide catalysts.

Metal oxides have been widely applied in chemical research and also exhibited great potential in e-NRR. To find the viability of electrocatalysts for catalyzing NH_3_ formation electrochemically at ambient conditions, 11 types of transition metal dioxides (NbO_2_, TaO_2_, RuO_2_, ReO_2_, TiO_2_, OsO_2_, RhO_2_, MnO_2_, CrO_2_, IrO_2_, and PtO_2_) in the rutile structure were investigated by DFT calculations on their (110) lattice planes [96]. The predicted onset potentials as a function of the binding energy of NNH were given in Figure 7c, with only two potential-determining steps. Among 11 types of transition metal dioxides, ReO_2_, TaO_2_, and OsO_2_, required an overpotential similar to, or lower than, the overpotential required for reducing nitrogen through nitrogensase, but that is believed to be approximately 0.63 V.

Moreover, the (110) facets of ReO_2_ and TaO_2_ were found to favor NNH adsorption over H adsorption, whereas IrO_2_ and NbO_2_ surfaces might be poisoned by adsorbed hydrogen atoms. Huang et al. [98] experimentally verified the potential of NbO_2_ nanoparticles as an efficient e-NRR electrocatalyst. Compared to Nb_2_O_5_ with a similar crystal structure but a different linkage style, the Nb^4+^ cation of NbO_2_ enabled effective N_2_ adsorption by proving empty d-orbitals and subsequent activation by back donation. Consequently, the NbO_2_ nanoparticles presented both an efficient NH_3_ production rate (11.60 µg h^−1^ mg_cat._^−1^) at −0.65 V and FE (32%) at −0.60 V, significantly higher than those of Nb_2_O_5_ nanoparticles under similar conditions.

Due to the industrial application of Fe-based catalysts in the Haber–Bosh process, Fe-based oxide materials were also considered as an efficient candidate in the field of e-NRR. Ever since nano-Fe_2_O_3_ was reported as an e-NRR electrocatalyst by Licht and co-workers, Fe-based oxides have attracted wide attention. Later, a complementary theoretical study demonstrated the chemical formation process of NH_3_ on two kinds of hematite (γ-Fe_2_O_3_) surfaces. Compared with single-iron (Fe–O_3_–Fe–), double-iron (Fe–Fe–O_3_–) needed a smaller applied bias for proton transfer, owing to the two available reactive Fe sites on this surface [99]. Kong et al. [7] firstly investigated the e-NRR activity of nanosized γ-Fe_2_O_3_ electrocatalysts at low temperature (<65 °C), in basic aqueous solution and in the membrane electrode assembly (MEA)-based reactors, respectively. Compared with the half-cell, the e-NRR activity in MEA-based reactors was observed with a dramatical increase to 55.90 nmol h^−1^ mg^−1^. The enhanced catalytic performance may be attributed to the efficient utilization of γ-Fe_2_O_3_ after it is coated on the porous carbon paper. Furthermore, the Fe_2_O_3_-CNT and oxygen-vacancy-enriched-Fe_2_O_3_/CNT catalysts were also reported [100,101]. In addition to Fe_2_O_3_, Fe_3_O_4_ was also reported to be catalytically active for e-NRR. A spinel Fe_3_O_4_ nanorod on a Ti mesh (Fe_3_O_4_/Ti) was fabricated as a catalyst for electrochemical N_2_ conversion to NH_3_, with long-term electrochemical durability [102]. Hu et al. [103] investigated the Fe-based materials for electrocatalytic NH_3_ production and revealed the effect of different chemical states of Fe on the e-NRR activity. The Fe/Fe_3_O_4_ catalyst was fabricated via in situ growth on the Fe foil. In particular, the activity and selectivity of Fe/Fe_3_O_4_ were superior to those of Fe, Fe_3_O_4_ and Fe_2_O_3_ nanoparticles. It has been concluded that the e-NRR catalytic performance was related to Fe/Fe oxide ratio.

Much attention has also been focused on developing Mo-based oxides as high-performance e-NRR electrocatalysts. Sun et al. [90] discovered that MoO_3_ nanosheets exhibited remarkable e-NRR activity with excellent selectivity in 0.10 M HCl electrolyte (NH_3_ yield: 29.43 μg h^−1^ mg_cat._^−1^ and FE: 1.9%). It was found that the outermost Mo atoms served as the active sites for effective N_2_ adsorption, by DFT calculations. To further tailor the performance of Mo oxides, a hybrid catalyst of MoO_2_ on reduced graphene oxide (MoO_2_/RGO) was fabricated to catalyze the e-NRR. In 0.10 M Na_2_SO_4_ electrolyte, an enhanced e-NRR performance was obtained, with an NH_3_ yield of 37.4 μg h^−1^ mg^−1^ and FE of 6.6% at the potential of −0.35 V (vs. RHE) [104]. Relative to MoO_2_ alone, MoO_2_/RGO hybrid promoted the electronic interactions with *N_2_H, and enabled the donation of more electrons from the active Mo sites to *N_2_H, leading to the enhanced e-NRR activity. Based on the vacancy and heterostructure engineering, O-vacancy-rich MoO_3-*x*_ anchored on Ti_3_C_2_T_x_-MXene (MoO_3-*x*_/MXene) was explored, as a highly efficient and selective e-NRR electrocatalyst, obtaining an exceptional e-NRR performance with an NH_3_ yield of 95.8 µg h^−1^ mg^−1^ at −0.4 V and a FE of 22.3% at −0.3 V [97]. MoO_3−x_/MXene produce steady NH_3_ yields and FEs during consecutive seven cycles of electrolysis, while just a very small change compared to the initial one. In Figure 7d, OV-rich MoO_3_ and MoO_3_/MXene achieved higher e-NRR activities with respect to their corresponding OV-rich MoO_3_ and MoO_3_/MXene, indicating the critical role of OVs for substantially improving e-NRR performance. Through in situ Raman spectroscopy adopted in a tailor-made electrolytic cell (Figure 7e), the 3D plots for the time-dependent Raman spectra traces of various catalysts at −0.4 V were shown in Figure 7f–i, to track the changes in surface chemical bonds of considerable catalysts. Together with molecular dynamics simulations and DFT computations, the synergistic effects of OVs and MXene on the e-NRR of MoO_3−x_/MXene were confirmed.

SnO_2_, known for its low cost and high chemical stability, was initially developed by Zhang et al. [105] as an e-NRR electrocatalyst in the form of cubic sub-micron SnO_2_ particles loaded on carbon cloth (SnO_2_/CC). However, to enhance conductivity and active sites of such catalyst, Chu et al. [106] developed a novel fluorine-doped SnO_2_ mesoporous nanosheets on carbon cloth (F-SnO_2_/CC) as an e-NRR electrocatalyst. From the calculations, F-doping contributed to readily enhance the conductivity and increase the positive charge density on active Sn sites, resulting in reduced reaction energy barriers and enhanced e-NRR activities. This group also investigated the e-NRR performance of supporting the ultrasmall SnO_2_ QDs on RGO [106]. Similarly, the experimental and theoretical results confirmed that coupling SnO_2_ QDs and RGO could readily tailor the electronic structure of SnO_2_, leading to fascinating e-NRR activity.

As one of the classical semiconductors, TiO_2_-based materials have been firstly investigated as efficient photocatalysts in the photo-reduction of N_2_ to NH_3_. Lately, Sun et al. [107] explored the TiO_2_ nanosheets array on the Ti plate (TiO_2_/Ti) for electrochemical N_2_ conversion to NH_3_. When measured in 0.10 M Na_2_SO_4_, TiO_2_/Ti achieved a high NH_3_ yield of 9.16 × 10^−11^ mol s^−1^ cm^−2^ with an FE of 2.50% at −0.70 V vs. RHE, due to the enhancement of adsorption and activation of N_2_ by in situ-generated oxygen vacancies. To further enhance electronic conductivity, a TiO_2_ nanoparticle-reduced graphene oxide hybrid (TiO_2_-rGO) was fabricated as an e-NRR electrocatalyst, by Sun’s group [108]. The FE of TiO_2_-rGO was enhanced to 3.30% at −0.90 V vs. RHE.

Inspired by the enhanced activity of nitrogenases with Mn^2+^, Wang et al. [109] reported MnO particles on Ti mesh (MnO/TM) as a robust e-NRR catalyst. In 0.10 M Na_2_SO_4_ electrolyte, such catalyst achieved a high FE up to 8.02% and a large NH_3_ production of 1.11 × 10^−10^ mol s^−1^ cm^−2^ at −0.39 V (vs. RHE). Theoretical calculations further revealed that the MnO(200) surface preferentially adsorbed N atoms instead of H atoms, and the potential-determining step was *N_2_ → *N_2_H transformation. Additionally, a spinel LiMn_2_O_4_ nanofiber could act as a noble-metal-free electrocatalyst for NH_3_ synthesis with an excellent FE of 7.44% [110], much higher than that of the previous Mn_3_O_4_ nanocube (3.00%) [111] and Mn_3_O_4_ nano-particles-reduced graphene oxide (3.52%) [112]. Besides, Cr_2_O_3_ nanofiber was fabricated as a non-noble-metal e-NRR electrocatalyst [113]. This catalyst achieved an efficient performance in both FE and NH_3_ formation, with favorable electrochemical durability.

## 5. Conclusions and Outlook

In conclusion, we discuss the recent advances of metal-based e-NRR electrocatalysts using the structure–function relationship, concluding that noble-metal-based catalysts, non-noble-metal-based catalysts and metal compound catalysts provide a fundamental basis for rational electrocatalyst design. Additionally, the challenges and prospects for e-NRR were proposed. Although the encouraging progress on e-NRR electrocatalysts has been achieved with favorable performance, the reported studies of e-NRR still have a long distance to go in contrast with the industrial Haber–Bosch process, from the point of view of industrialization and commercialization. Research in e-NRR still faces several key challenges in the near future.

(1) Selectivity of catalysts is a much larger issue for improving FE, due to the competitive reactions. The hydrogen and hydrazine simultaneously generated during the ammonia production resulted in a relatively low selectivity towards e-NRR [114,115,116]. The designed catalysts are required to have a much stronger binding energy of *N compared to the *H. In addition, the strategy of enhancing the solubility of N_2_ in the electrolyte also needs to be developed [117,118,119,120].
(2) In-depth studies of the e-NRR mechanism are still limited and plain. Most of the research only simulated the possible reaction pathways and the energy barriers using theoretical calculations. Most reported theoretical studies for identifying the research direction toward electrocatalyst design were performed on appropriate and simplified models. However, the real-time operation of e-NRR is always in combination with different reaction conditions and parameters (pH, environmental electrolyte, voltage, environmental temperature, and pressure, etc.) that ought to be considered in further calculations [3,28,121].
(3) The stability of the catalyst is as important as catalyst activity and selectivity. After longtime electrolysis operation, the electrocatalyst may undergo decomposition and deactivation [4,122]. Therefore, the electrocatalysts should be designed with a stable structure. Additionally, the prolonged periods for the stability tests are recommended to screen active electrocatalysts for e-NRR [28,123].
(4) The relationship between structure and activity for e-NRR is of significance to provide a guideline on the rational design of novel catalysts. Despite the great efforts on developing advanced materials for e-NRR, it remains challenging to reveal the relationship between the structure and activity under the reaction conditions [124,125]. In situ analytical techniques and theoretical experiments are highly desirable and beneficial in providing evidence of catalyst surface reconstruction and generation of key intermediates under real-time reaction conditions, as well as in achieving a comprehensive understanding of the kinetic mechanism [25,124,126,127].

## Data Availability

Not applicable.
